# Lockdown stringency and paediatric self-harm presentations during COVID-19 pandemic: retrospective cohort study

**DOI:** 10.1192/bjo.2022.41

**Published:** 2022-03-24

**Authors:** Ben Hoi-ching Wong, Mehrak Vaezinejad, Paul L. Plener, Tauseef Mehdi, Liana Romaniuk, Elizabeth Barrett, Haseena Hussain, Alexandra Lloyd, Jovanka Tolmac, Manish Rao, Sulagna Chakrabarti, Sara Carucci, Omer S. Moghraby, Rachel Elvins, Farah Rozali, Ereni Skouta, Fiona McNicholas, Benjamin Baig, Dejan Stevanovic, Peter Nagy, Chiara Davico, Hassan Mirza, Evren Tufan, Fatima Youssef, Ben Meadowcroft, Dennis Ougrin

**Affiliations:** King's College London, Child and Adolescent Psychiatry, London, UK; South London and Maudsley Mental Health NHS Trust, London, UK; Medical University of Vienna, Vienna, Austria, and Department of Child and Adolescent Psychiatry and Psychotherapy, University of Ulm, Ulm, Germany; Berkshire Healthcare NHS Foundation Trust, Bracknell, Bracknell Forest, UK; The University of Edinburgh Centre for Clinical Brain Sciences, Edinburgh, UK; Temple Street Children's University Hospital, Dublin, Ireland; Hertfordshire Partnership University NHS Foundation Trust, Child and Adolescent Mental Health Services, Hatfield, Hertfordshire, UK; Hertfordshire Partnership University NHS Foundation Trust, Child and Adolescent Mental Health Services, Hatfield, Hertfordshire, UK; Central and North West London NHS Foundation Trust, London, UK; South London and Maudsley Mental Health NHS Trust, London, UK; South London and Maudsley Mental Health NHS Trust, London, UK; Facoltà di Medicina e Chirurgia, Università degli Studi di Cagliari, Sardegna, Italy; South London and Maudsley Mental Health NHS Trust, London, UK; Manchester University NHS Foundation Trust, Greater Manchester, UK; NHS Lothian, Edinburgh, UK; NHS Lothian, Edinburgh, UK; University College Dublin School of Medicine, Dublin, Ireland; South London and Maudsley Mental Health NHS Trust, London, UK; Clinic for Neurology and Psychiatry for Children and Youth, Belgrade, Serbia; Vadaskert Child and Adolescent Psychiatric Hospital, Vadaskert, Hungary; Universita degli studi di Torino, Italy; Sultan Qaboos University, Muscat, Oman; Abant Izzet Baysal University Medical Faculty, Bolu, Turkey; Dubai Department of Medical Education, Dubai, United Arab Emirates; NHS Lothian, Edinburgh, UK; King's College London, Child and Adolescent Psychiatry, London, UK

**Keywords:** Self-harm, lockdown, children, adolescent, psychiatric emergency, COVID-19, lockdown stringency, retrospective study

## Abstract

**Background:**

Lockdown during the pandemic has had significant impacts on public mental health. Previous studies suggest an increase in self-harm and suicide in children and adolescents. There has been little research on the roles of stringent lockdown.

**Aims:**

To investigate the mediating and predictive roles of lockdown policy stringency measures in self-harm and emergency psychiatric presentations.

**Method:**

This was a retrospective cohort study. We analysed data of 2073 psychiatric emergency presentations of children and adolescents from 23 hospital catchment areas in ten countries, in March to April 2019 and 2020.

**Results:**

Lockdown measure stringency mediated the reduction in psychiatric emergency presentations (incidence rate ratio of the natural indirect effect [IRR^NIE^] = 0.41, 95% CI [0.35, 0.48]) and self-harm presentations (IRR^NIE^ = 0.49, 95% CI [0.39, 0.60]) in 2020 compared with 2019. Self-harm presentations among male and looked after children were likely to increase in parallel with lockdown stringency. Self-harm presentations precipitated by social isolation increased with stringency, whereas school pressure and rows with a friend became less likely precipitants. Children from more deprived neighbourhoods were less likely to present to emergency departments when lockdown became more stringent,

**Conclusions:**

Lockdown may produce differential effects among children and adolescents who self-harm. Development in community or remote mental health services is crucial to offset potential barriers to access to emergency psychiatric care, especially for the most deprived youths. Governments should aim to reduce unnecessary fear of help-seeking and keep lockdown as short as possible. Underlying mediation mechanisms of stringent measures and potential psychosocial inequalities warrant further research.

Self-harm is the second leading cause of death in young people aged 15–29 years^1^ and is associated with significantly higher risk of completed suicide in children and adolescents. According to the National Institute for Health and Care Excellence (NICE), self-harm is defined as intentional self-injury or self-poisoning, regardless of motive or extent of suicidal intent.^2^ Longitudinal studies and cohort studies in the past two decades have suggested an upward trend in self-harm incidence in adolescents.^3^ During the coronavirus disease 2019 (COVID-19) pandemic, rigorous lockdown measures globally are likely to have had substantial impacts on psychological stress^4^ and self-harm in children and adolescents.^5^ As of summer 2021, many of these measures have been maintained in various countries in response to waves of infection. It is important to reflect on how self-harm has been affected by these measures and improve mental health service planning under lockdown measures in the future. Increases in anxiety and psychological distress in adolescents have been reported.^6^ School closures may have led to greater urge to self-harm, complicated by increased overthinking and negative coping strategies when staying at home.^7^ Overcrowding of living space may increase interpersonal stress within families, which is highly correlated with self-harm in adolescents.^8^ Increases in numbers and severity of incidents of domestic violence have been reported worldwide during lockdown.^9^ Despite improvements in understanding and treatment of self-harm, help-seeking is rare in young people who self-harm^10^ and might have further decreased owing to lack of privacy and fear of contracting COVID-19.^7,11^ Disruptions and restructuring of youth welfare and hospital services have been observed worldwide.^12^ Those who have severe mental illnesses are more exposed to such disruptions. Multiple studies have also raised concerns about inequalities, with some children struggling more than others. For example, the extent and intensity of community support networks in deprived regions were lowest during lockdown.^13^ Higher mobility contraction was found in more socioeconomically deprived regions.^14^ Despite increased exposure to these theoretical risk factors, to date, there has been no systematic quantitative research on how self-harm in children and adolescents varied with changes in lockdown measures. A potential increase in the child suicide rate in England in April and May 2020 compared with 2019 was unconfirmed statistically owing to the small number of cases.^15^ A recent international cohort study observed conflicting changes based on hospital psychiatric emergency presentations in ten countries.^16^ Emergency departments are usually the first point of contact into the mental health service system for youths who have mental health problems, especially self-harm. Fewer children and adolescents presented for psychiatric problems or self-harm in 2020 compared with 2019. It is difficult to ascertain the exact mechanism involved in these studies, as the results could reflect the consequences of the COVID-19 outbreak, lockdown measures, or both. Moreover, not all countries achieved complete lockdown at all times. Studies across the globe have suggested that stringency effectively reduced mobility in the community.^17^ Referrals to child and adolescent mental health services displayed various patterns in different periods of the lockdown.^18^ It is possible that the unintended consequences of lockdown varied with the magnitude of lockdown stringency, creating barriers to emergency presentations. Self-harm attempts and thoughts were found to have increased during the first 6 weeks of lockdown among young adults (18–29 years old) who were female, socially disadvantaged, from ethnic minority groups or had pre-existing mental disorders.^19^ One may wonder whether similar patterns were replicated in children and adolescents. This study aimed to investigate the potential roles of stringency as a mediator of and predictor for the changes in self-harm and psychiatric emergency presentations in children and adolescents during the lockdown in the first wave of COVID-19.

## Method

### Study design and population

This was a retrospective cohort study. The target cohort was children and adolescents under 18 years old who presented at the included emergency units with mental health emergencies, including self-harm, during March and April of 2020 and the same period in 2019. Data from both years were used for the mediation analyses. Subsample analyses of the effects of stringency on self-harm characteristics during lockdown used only self-harm presentations from 2020.

### Data sources

Data were extracted from electronic patient records of hospital emergency departments of 23 catchment areas in ten countries, and aggregate measures were constructed for 14 sites which were defined based on similarities of sociodemographic characteristics and geographic proximity. This choice of observation unit maximises statistical power by combining catchment areas with small numbers of presentations, while allowing the presence of multiple areas within some countries and preserving potential within-country differences. Locations of emergency units and categorisation can be found in the Supplementary material available at https://doi.org/10.1192/bjo.2022.41. The emergency departments included serve a total of around 6.5 million children and adolescents, receiving around 200 000 paediatric emergency presentations per year. The catchment areas were from a mixture of developed high-income countries (England, Scotland, Austria, Hungary, Ireland and Italy), developing high-income countries (Oman and the United Arab Emirates) and developing middle-income countries (Serbia and Turkey), representing a variety of healthcare systems.

A stringency index of lockdown measures for each of the ten countries was extracted from the Oxford COVID-19 Government Response Tracker (OxCGRT) for March and April 2020.^20^ Stringency in 2019 was taken as zero owing to the absence of any lockdown measure in the participating countries. OxCGRT is a tracker collecting systematic cross-national information of government responses daily during the full period of COVID-19 spread. A standardised composite index of stringency for each country is created daily based on aggregated scores for nine policy response indicators: school closure, workplace closure, cancellation of public events, restrictions of gatherings, closure of public transport, stay-at-home requirements, restrictions on internal movement, international travel controls and public information campaigns. Further information on indicator coding and score calculation can be found in the paper by Hale et al.^20^ Although the tracker does not distinguish between nations within the UK, there was a high level of policy coordination across England and Scotland throughout March and April 2020.^21^ Therefore, the current study applied the same UK stringency index for all sites in England and Scotland.

### Outcomes

#### All psychiatric emergency presentations

For each psychiatric emergency presentation, the following sociodemographic information was collected: sex; age; whether the individual was in the local dominant ethnic group; whether the individual was in education, employment or training; postcode of address; whether the individual was looked after by the local authority; and whether the young person lived with both their biological parents. The most recent records of deprivation deciles were collected only from England's and Scotland's governments^22,23^ and matched to presentations based on the full postcode of the accommodation. Deprivation deciles are ranked from the most deprived 10% (1st decile) to the least deprived 10% (10th decile). Each hospital presentation was coded with respect to whether it was due to self-harm according to the definition of the NICE guidelines.^2^ This covers non-suicidal self-injury, non-suicidal self-poisoning, attempted suicides, and self-harm with unclear or mixed intent.

#### Subsample of self-harm presentations

Whether a self-harm presentation was severe was defined as meeting at least one of the following criteria: (a) involved a high-lethality self-harm method (including hanging, drowning, jumping from heights, using a firearm, potentially lethal self-poisoning dose or choice of poison, and self-injury involving major vessels); (2) any self-harm resulting in admission to an intensive care unit; (3) any self-harm resulting in admission to an acute ward for medical reasons, with admission lasting for 72 h or more.

Other self-harm characteristics included: whether self-harm was performed with suicidal intent, whether a violent method (firearm, hanging, drowning or jumping from heights) of self-harm was used, whether alcohol use was involved in self-harm, whether drug use was involved in self-harm, and whether social media was used to communicate or broadcast self-harm. Relevant self-harm history included: whether the individual had presented at hospital for self-harm in the previous year, whether the individual had previously self-harmed in the community and whether any family member had a history of self-harm.

Coders could record the presence of up to three clinical diagnoses: emotional disorders, behavioural disorders, psychotic disorders, eating disorders, neurodevelopmental disorders, substance misuse disorders, somatoform disorders and personality disorders. Up to three precipitants could also be recorded: row with a family member, row with a friend, row with a boyfriend or girlfriend, social isolation, and school pressure.

### Statistical analysis

Summary statistics of sociodemographic information and site-specific sample size were provided for all psychiatric emergency presentations. All statistical analyses in the current study were performed on Stata/MP 16 software.^24^

### Mediation analyses

**Fig. 1:**
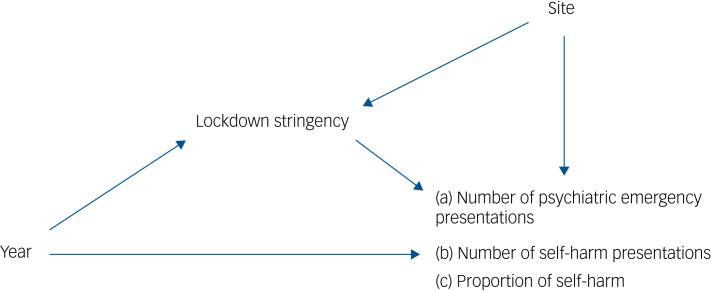
Hypothesised mediation pathway.

All presentations from both years were used. Mediation analyses were performed using the paramed command on Stata 16,^25^ as illustrated in Fig. 1. Lockdown stringency was hypothesised to mediate the effect of year on presentations. The outcome variables of interest were: (a) number of psychiatric emergency presentations per site; (b) number of self-harm presentations per site; and (3) a binary variable indicating self-harm presentations. A negative binomial regression model was specified for the former two outcomes. This allows for potential overdispersion due to repeated presentations by the same children and adolescents, or intra-site differences from catchment area clustering. Logistic regression was used to model the proportion of self-harm presentations in all presentations. The 14 sites included in each year period (2019 and 2020) were coded in 28 orthogonal contrasts and entered as confounders in paramed. This provided an approximation of a random effects model that utilised within-site variability, adjusting for the correlation between repeated presentations and repeated stringency values within each year period at each site.

Mediation effects and their 95% confidence intervals were estimated with 1000 bootstrap samples. In each analysis, the natural indirect effect (NIE) captured the effect of year on outcomes via the mediation of stringency; a significant NIE would suggest the presence of an underlying mediation mechanism. Bootstrapping only estimated the controlled direct effect (CDE) of year, and no natural direct effect was produced. CDE is often of greater interest in evaluating policy interventions.^26^ In the current study, CDE informed whether there was any direct pathway of year effect outside the effect of stringency. Total effect was estimated to summarise the resulting overall year effect.

### Analyses of the effect of lockdown measures on self-harm presentations during lockdown

We investigated the effect of the stringency of the country's lockdown measures on various characteristics of emergency presentations for self-harm. Only self-harm presentations in March and April 2020 were included in these analyses. Generalised estimating equations (GEE) models were fitted to estimate the stringency effects on age and all binary outcomes (sociodemographic characteristics, self-harm characteristics and relevant history, clinical diagnoses, and precipitating factors). GEE is a population-averaged approach that has the advantage of providing robust statistical inference in multilevel modelling.^27^ Exchangeable correlation structure was selected to account for repeated presentations and within individuals. Using robust standard errors and allowing intra-subject correlations, an ordered logistic regression model was fitted for the stringency effect on deprivation deciles of presentations. Marginal effects for each decile were plotted to visualise variation across deprivation levels. Site was included as a second explanatory variable in all our models to account for other site differences.

All estimates for stringency effects in self-harm presentations during lockdown were reported for each ten-unit increment in stringency index. This does not alter the *P*-values of results and produces proportionally magnified estimates and confidence intervals. As stringency index is a standardised measure ranging from 1 (no restriction) to 100 (complete restriction), each unit of change in stringency represents almost negligible differences in lockdown policies. In reality, much greater changes are expected every time governments introduce or withdraw restriction measures. For reference, the stringency index in March to April 2020 for each of the ten included countries had standard deviations ranging from 34.1 to 46.8. The odds ratios (ORs) for each unit increase of stringency might be extremely small numerical values that are susceptible to rounding errors and are not useful representations of the effects in practice. Missing data were assumed to occur completely at random, and complete case analyses were adopted.

### Ethical approval and consent to participate

This study was based on data from the National Commissioning Data Repository (NCDR) obtained under licence from the UK Medicines and Healthcare Products Regulatory Agency. The study was approved by the King's College London/South London and Maudsley NHS Foundation Trust service evaluation and clinical audit committee (ref no. AP1312/05/2020).

## Results

### Descriptive statistics of all psychiatric emergency presentations

A total of 2073 psychiatric emergency presentations from 1795 unique children and adolescents (mean age = 14.9 years) were eligible, with 834 presentations in 2020. Psychiatric emergency presentations at all sites were mostly from females (67.5% female, 30.7% male and 1.8% other) and predominantly from the dominant ethnic group (73%). The mean deprivation decile for England and Scotland presentations was 5.5. Among 1352 presentations with relevant data, 12% of children and adolescents were looked after by the local authority. Among those who were not looked after, 41% lived with both biological parents. Eighty-nine per cent of presentations with available information were in education, employment or training.

### Mediation effect of stringency on psychiatric emergency presentations

Increase in stringency significantly mediated the reduction in psychiatric emergency presentations in 2020 (incidence rate ratio of the NIE [IRR^NIE^] = 0.41, 95% CI [0.35, 0.48]). Conversely, after controlling for stringency, there was a significantly increased rate of psychiatric emergency presentations in 2020 compared with 2019 (IRR^CDE^ = 1.37, 95% CI [1.10, 1.78]). However, the positive direct effect of year was masked by the mediation effect of stringency. The overall incidence of psychiatric emergency presentations in children and adolescents was significantly reduced in 2020 compared with 2019 (IRR of the total effect [IRR^TE^] = 0.56, 95% CI [0.48, 0.69]).

### Mediation effect of stringency on self-harm emergency presentations

Among all emergency presentations, stringency significantly mediated the indirect reduction effect of year on number of self-harm presentations (IRR^NIE^ = 0.49, 95% CI [0.39, 0.60]). The CDE of year was not significant after controlling for stringency (IRR^CDE^ = 1.41, 95% CI [0.38, 5.36]). This was again masked by the mediated NIE. The number of self-harm presentations in children and adolescents did not significantly decrease in 2020 (IRR^TE^ = 0.69, 95% CI [0.19, 2.55]).

The indirect effect of year on proportion of self-harm presentations via mediation of stringency level was positive and significant (OR^NIE^ = 1.36, 95% CI [1.02, 1.81]). The direct effect of year after controlling for stringency was not significant (OR^CDE^ = 3.97, 95% CI [0.69, 32.32]). Altogether, the proportion of self-harm presentation increased significantly in 2020 (OR of the total effect [OR^TE^] = 5.39, 95% CI [1.04, 38.79]).

### Subsample analyses of self-harm presentations during lockdown (*n* = 470)

#### Characteristics and clinical profiles

The mean age (15.3 years) of self-harm presentations during lockdown did not change significantly with stringency (*b* = 0.0410, 95% CI [−0.002, 0.084], *P* = 0.06). Analyses for other characteristics are summarised in Table 1. The proportion of male children presenting with self-harm increased with stringency (estimated 8% increase per ten units increase of stringency index). Looked after children constituted a greater proportion of self-harm presentations when lockdown became more stringent (OR = 1.12 per ten-unit increment of stringency index). There was no significant effect of stringency on proportions of severe self-harm, suicidal intent, or other self-harm characteristics and relevant history. No particular psychiatric diagnosis in self-harm presentations during lockdown was associated with change in stringency. The proportion of ‘row with a friend’ as a precipitant decreased significantly when stringency increased (OR = 0.87 per ten-unit increment of stringency). A similar reduction was found for proportion of ‘school pressure’ as precipitant, with an estimated 16% decrease in odds when the stringency index increased by ten units. Conversely, the proportion of ‘social isolation’ as precipitant of the self-harm presentation increased by an estimated 15% for each ten-unit increase in stringency. Stringency had no statistically significant effect on the proportions of self-harm presentations where the precipitant was ‘row with a family member’ or ‘row with a boyfriend or girlfriend’.

**Table 1:** Estimates of stringency effects on self-harm presentations in lockdown (*n* = 470)

	Proportion (available sample size)	Effect estimates (per ten-unit increment of stringency index)		
		OR	95% CI for OR	*P*-value
Sociodemographic characteristics				
Female	75% (462)	0.93	[0.87, 0.996]	0.039
Male	23% (462)	1.08	[1.01, 1.16]	0.026
Dominant ethnicity	74% (375)	1.07	[1.00, 1.16]	0.061
In EET	88% (306)	0.97	[0.87, 1.08]	0.56
Looked after children	13.2% (325)	1.12	[1.003, 1.25]	0.044
Parents live together	42% (235)	0.95	[0.88, 1.03]	0.247
Self-harm characteristics and history				
Severe self-harm	19.4% (469)	1.07	[0.99, 1.16]	0.104
Suicidal intent	55% (435)	1.01	[0.95, 1.08]	0.67
Violent method of self-harm	7.6% (461)	1.12	[0.99, 1.27]	0.076
Alcohol involved in self-harm	10.0% (372)	0.94	[0.84, 1.05]	0.287
Drug involved in self-harm	7.0% (371)	1.01	[0.87, 1.17]	0.91
Social media used to communicate self-harm	8.0% (302)	1.02	[0.88, 1.18]	0.81
Self-harm history in community	81% (341)	0.99	[0.90, 1.09]	0.87
Self-harm presentation in previous year	47% (324)	1.05	[0.98, 1.13]	0.158
Family history of self-harm	18.4% (196)	1.01	[0.89, 1.15]	0.86
Clinical diagnosis				
Emotional disorders	66% (384)	1.00	[0.93, 1.08]	0.99
Behavioural disorders	14.3% (384)	1.10	[0.99, 1.21]	0.076
Psychotic disorders^a^	2.60% (384)	N/A	N/A	N/A
Eating disorders^a^	3.65% (384)	N/A	N/A	N/A
Neurological disorders	15.9% (384)	1.06	[0.96, 1.16]	0.241
Substance use disorders	6.8% (384)	1.06	[0.93, 1.20]	0.393
Somatoform disorders^a^	2.08% (384)	N/A	N/A	N/A
Personality disorders	14.1% (384)	0.99	[0.90, 1.09]	0.84
Precipitating factor				
Row with a family member	37.7% (308)	0.99	[0.92, 1.08]	0.90
Row with a friend	11.4% (308)	0.87	[0.77, 0.99]	0.029
Row with a boyfriend or girlfriend	10.1% (308)	1.13	[0.99, 1.28]	0.072
Social isolation	16.9% (308)	1.15	[1.04, 1.27]	0.008
School pressure	13.3% (308)	0.84	[0.74, 0.94]	0.003

EET, education, employment or training; N/A, not available.

a. Excluded from analysis owing to low counts of events.

#### Variation across deprivation levels

**Fig. 2:**
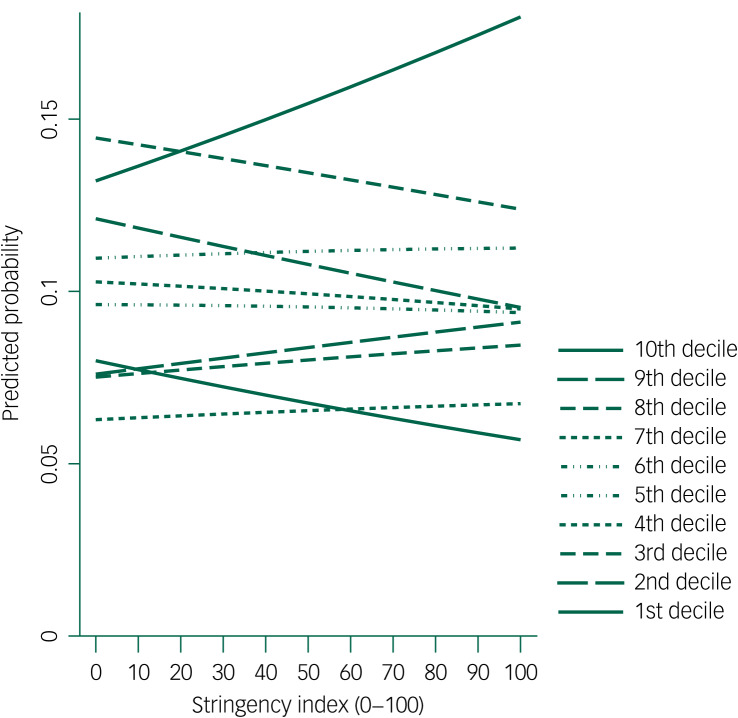
Stringency effect on predicted probability of self-harm presentations from each deprivation decile (1st decile = most deprived, 10th decile = least deprived).

Ordered logistic regression model was fitted for self-harm presentations in England and Scotland. Stringency did not significantly predict the deprivation deciles of presentations (OR = 1.04, 95% CI [0.97, 1.11], *P* = 0.236). The estimated probability for each decile in self-harm presentations during lockdown (Fig. 2) revealed two distinct directions for the less deprived half (6th to 10th deciles) and the more deprived half (1st to 5th deciles) of deprivation levels. The more stringent the restriction measures, the more likely it was that self-harm presentations were made by children and adolescents from the less deprived half, and the less likely it was that they were from the more deprived half. In maximum lockdown situations, self-harm presentations were most likely to be from the least deprived decile and least likely to be from the most deprived decile. However, less deprived deciles (e.g. the 6th decile) did not always have higher predicted probability than relatively more deprived deciles (e.g. the 3rd decile) for self-harm presentations in our sample. This potentially explains the overall non-significant estimates in the ordered model.

## Discussion

### Main findings

To the best of our knowledge, this is the only quantitative study to date that explores the effects of COVID-19 lockdown stringency on self-harm in children and adolescents. Considering data from diverse healthcare systems in ten countries, our findings suggested that lockdown stringency mediated the reduction in numbers of psychiatric emergency mental health presentations and specifically self-harm presentations in children and adolescents, and mediated the positive relationship between year and proportion of self-harm presentations. When lockdown became more stringent, increased self-harm presentations were evident in males, looked after children and those who reported social isolation as a precipitant.

### Comparison with previous studies

Stringent lockdown may have a mixture of positive and negative effects on self-harm in children and adolescents.^28^ In light of previous studies of risk factors in lockdown, stringent measures might have introduced unintended barriers to children and adolescents presenting at hospital emergency units. Recent studies suggested that mobility in the general public reduced with increase in stringency.^17^ School closure was found to be associated particularly with time staying at home. While youths who self-harm may avoid presentations at hospitals out of fear of contracting and spreading the disease or concerns of being labelled as an ‘attention-seeker’,^11^ stringent government restrictions might have inadvertently further discouraged help-seeking behaviour in those who were in need. Moreover, school closures reduced pathways to hospital presentations, as psychological support and gatekeeping are critical roles of schools.

Another possible explanation may provide more optimism. Increased stringent restrictions from governments might have surprising protective effects against acute psychiatric problems and self-harm in children and adolescents. Stringent lockdowns potentially reduced exposure to certain stressors and anxiety-provoking social gatherings. Victimisation doubles the risk of self-harm behaviour and thought in adolescents,^29^ and school is the major setting where face-to-face bullying takes place. Fewer social contacts may also result in fewer interpersonal conflicts with friends, and exposure to peer self-harm, both of which are important risk factors for adolescent self-harm.^1^ This corresponds to the reduced odds of self-harm precipitated by conflicts with friends when lockdown stringency increased. The relieved academic pressure during lockdown may contribute to better well-being. Individual learning at home may be beneficial to youths with hyperkinetic disorders or other learning problems.^28^ These echo the observed decrease in proportion of self-harm presentations precipitated by a row with a friend or school pressure when lockdown became more stringent. Children may benefit from improved supervision and quality time with parents, hence potentially reducing the opportunity or need to engage in maladaptive self-harm coping strategies. During lockdown, some parents might have had more free time to interact with their children and show more empathy, leading to better family connectedness, which is of high importance in preventing self-harm in adolescents.^30^ Stay-at-home requirements may increase opportunities for aggression to be managed promptly by carers, reducing another common reason for psychiatric emergency presentations.

However, it is likely that not all children benefited from these positive effects, especially in families facing stress, unemployment, overcrowding or domestic abuse.^8^ There might not be sufficient positive peer contacts to offset such familial toxicity. In our study, social isolation precipitated a larger proportion of self-harm presentations when stringency increased, possibly owing to a lack of meaningful social interactions outside families.^12^ Increase in stringency did not seem to increase the proportion of severe self-harm among all self-harm presentations, with no significant change in fatal methods or presence of suicide intent. This mirrors Japanese reports of no change in child suicide rates during school closures.^31^ Stringent measures seemed to affect certain groups more than others. Previous research warned about increased risks of self-harm and suicide in vulnerable youths, such as those in foster care.^32^ Our study replicated these findings, as evidenced by an increase in the proportion of looked after children in self-harm presentations as stringency increased. In contrast to the higher self-harm rate in young female adults,^19^ the odds of a self-harm presentation being from a male child or adolescent increased with stringency, although the underlying reason for this was unclear.

The distinct patterns in predicted probability of self-harm presentations across deprivation levels warrant attention. Lockdown measures potentially presented greater barriers to accessing emergency psychiatric services for children and adolescents from the more deprived half. This potentially reinforces a recent finding that mobility contractions are higher in areas of lower income *per capita* and higher inequality.^14^ Children from economically deprived neighbourhoods generally have poorer access to mental health services.^33^ Their families are more likely to be unemployed in this financially difficult period, and to have more negative psychological outcomes and less social capital.^13^ We are aware that inequalities exist in medical morbidity and mortality from COVID-19, disproportionately affecting those from minority groups.^34^ Our results flagged a possible widening of psychosocial inequalities as a result of lockdown measures, although the exact association cannot be determined, and conclusions should not be drawn disproportionately.

### Implications for policies and research

A major clinical implication of our study is the need for a better healthcare pathway to reach and support those with severe mental health needs. Previous studies have suggested that at least half of adolescents who self-harm have no consequent contact with medical or psychological services.^35^ Help-seeking is likely to be worsened amid stringent lockdown restrictions. Policy makers and commissioners should prioritise funding and development of intensive community mental health services and telepsychiatry to provide assessments and interventions outside hospitals.^36^ Stringent physical distancing policies should be as short-lasting as possible to avoid build-up of psychiatric risks. Governments are recommended to provide a clear rationale for the measures to avoid public confusion, fear and misconception.^5^ Meanwhile, reducing social isolation in children and adolescents while maintaining physical distancing is a challenging yet crucial target in service planning and technology development. Consideration needs to be given to protecting youths and families who might be most adversely affected by lockdown policies.

Building on current findings, future research may explore specific components of policy stringency, such as school closures. There are concerns around psychosocial inequalities. It is important to understand the variation in psychological outcomes when lockdown stringency increases from the perspectives of different socioeconomic statuses. Looked after children and males were found to be overrepresented in self-harm presentations, but further research is needed to evaluate whether these are populations that receive more support than others, or at-risk groups that services need to prioritise.

### Limitations and strengths

The current study was limited by its observational nature. Our findings can only be used to complement theoretical underlying mediation effects instead of concluding the definitive causal mechanism. Our mediation models were not inherently multilevel and might be susceptible to influences of repeated presentations or stringency values. Second, our data were extracted from local emergency unit records. Some presentations may have been unrecorded, especially during the COVID-19 pandemic when most healthcare systems risked being overloaded. Our findings may also be an inaccurate reflection of self-harm in the community. For example, the proportion of severe self-harm is most likely to be magnified at emergency units. Data completion is poor for certain variables, especially for rare conditions such as psychotic disorders. We did not have access to catchment area population for weighted analyses or data beyond the first 2 months of lockdown. In addition, the current study did not investigate the differentiation in responses in each country. Cultural differences could be an interesting subject regarding the effects of stringency or willingness to adhere. Despite these limitations, the current study is the first to consider the impact of lockdown stringency on self-harm presentations. Our mediation models separated changes contributed by the lockdown measures from changes resulting from the pandemic. We used a large sample from ten countries, with site differences being accounted for in analyses. This provided greater statistical power to detect potential effects. Our findings may have high generalisability across cultures and different healthcare systems.

The current study is the first to date to address this gap, using an international sample of over 2000 children and adolescents in ten countries and a validated standardised measure of lockdown stringency. We showed that (a) the reductions in numbers of psychiatric emergency presentations and self-harm presentations were mediated by lockdown stringency; (b) lockdown measures masked the potential increase in these numbers in 2020 (probably due to other COVID-19-related factors); (c) lockdown relieved school pressure and peer conflicts while exacerbating social isolation; and (d) socioeconomic inequality possibly widened, with populations of deprived neighbourhoods accessing psychiatric emergency services less frequently when lockdown became more stringent. Our findings raise further important questions, such as the effects of particular aspects of lockdown measures and ways they hamper help-seeking or reduce psychiatric risks. Further studies are needed to improve psychiatric service engagement for children and adolescents in the future.

## Data Availability

Electronic health records are, by definition, considered ‘sensitive’ data in the UK by the GDPR and cannot be shared via public deposition because of information governance restrictions in place to protect patient confidentiality. Access to data is possible only once approval has been obtained through the individual constituent entities controlling access to the data.

## Supplementary material

For supplementary material accompanying this paper visit https://doi.org/10.1192/bjo.2022.41. click here to view supplementary material
