# Supplementary material for: Lockdown stringency and paediatric self-harm presentations during COVID-19 pandemic: retrospective cohort study
Source: BJPsych Open. 2022 Mar 24;8(2):e75. doi: 10.1192/bjo.2022.41 (PMC8963968; doi:10.1192/bjo.2022.41)
Supplement: Supplementary file 1 [file bjosup.zip › S2056472422000412sup002.docx]

**Supplementary material 2**

Stata output example

. paramed countSH, avar(yearC) mvar(stringency) cvars(dsite2 dsite3 dsite4 dsite5 dsite6 dsite7 dsite8 dsite9 dsite10 dsite11 dsite12 dsite13 dsite14 /

> *

> */ dsite2_2020 dsite3_2020 dsite4_2020 dsite5_2020 dsite6_2020 dsite7_2020 dsite8_2020 dsite9_2020 dsite10_2020 dsite11_2020 dsite12_2020 dsite13_202

> 0 dsite14_2020)/*

> */ a0(-0.5) a1(0.5) m(1) yreg(negbin) mreg(linear) nointer boot reps(1000) seed(1234)

Fitting Poisson model:

Iteration 0: log likelihood = -1588.9129

Iteration 1: log likelihood = -1493.96

Iteration 2: log likelihood = -1493.4051

Iteration 3: log likelihood = -1493.4048

Iteration 4: log likelihood = -1493.4048

Fitting constant-only model:

Iteration 0: log likelihood = -1863.0505

Iteration 1: log likelihood = -1847.74

Iteration 2: log likelihood = -1842.1347

Iteration 3: log likelihood = -1842.1243

Iteration 4: log likelihood = -1842.1243

Fitting full model:

Iteration 0: log likelihood = -1652.9093

Iteration 1: log likelihood = -1506.166

Iteration 2: log likelihood = -1476.1027

Iteration 3: log likelihood = -1473.4804

Iteration 4: log likelihood = -1473.4583

Iteration 5: log likelihood = -1473.4583

Negative binomial regression Number of obs = 1,708

LR chi2(28) = 737.33

Dispersion = mean Prob > chi2 = 0.0000

Log likelihood = -1473.4583 Pseudo R2 = 0.2001

------------------------------------------------------------------------------

countSH | Coef. Std. Err. z P>|z| [95% Conf. Interval]

-------------+----------------------------------------------------------------

yearC | .3422006 .1536472 2.23 0.026 .0410576 .6433435

stringency | -.0110368 .0017315 -6.37 0.000 -.0144305 -.0076432

dsite2 | -1.298372 .1578017 -8.23 0.000 -1.607658 -.9890864

dsite3 | .2527447 .1105718 2.29 0.022 .036028 .4694615

dsite4 | -1.108028 .1497246 -7.40 0.000 -1.401483 -.8145731

dsite5 | -.8772965 .1406597 -6.24 0.000 -1.152984 -.6016087

dsite6 | -.7057748 .1352876 -5.22 0.000 -.9709336 -.440616

dsite7 | -1.904125 .21956 -8.67 0.000 -2.334454 -1.473795

dsite8 | -2.336191 .2574947 -9.07 0.000 -2.840871 -1.831511

dsite9 | -.8887341 .1432913 -6.20 0.000 -1.16958 -.6078884

dsite10 | -3.093951 .3363018 -9.20 0.000 -3.75309 -2.434811

dsite11 | -2.340406 .2475237 -9.46 0.000 -2.825543 -1.855268

dsite12 | -3.112499 .336224 -9.26 0.000 -3.771486 -2.453512

dsite13 | -2.415001 .2510344 -9.62 0.000 -2.907019 -1.922982

dsite14 | -3.358615 .4119038 -8.15 0.000 -4.165931 -2.551298

dsite2_2020 | .0359274 .3156035 0.11 0.909 -.582644 .6544988

dsite3_2020 | .1724964 .2211436 0.78 0.435 -.2609371 .60593

dsite4_2020 | -.1039505 .2994492 -0.35 0.728 -.6908602 .4829591

dsite5_2020 | -.1863551 .2813193 -0.66 0.508 -.7377309 .3650206

dsite6_2020 | .5404704 .2705752 2.00 0.046 .0101527 1.070788

dsite7_2020 | .1573804 .43912 0.36 0.720 -.7032789 1.01804

dsite8_2020 | .5769553 .5149893 1.12 0.263 -.4324052 1.586316

dsite9_2020 | .6017 .2865825 2.10 0.036 .0400086 1.163391

dsite10_2020 | .0830875 .6726036 0.12 0.902 -1.235191 1.401366

dsite11_2020 | .2038829 .4950474 0.41 0.680 -.7663922 1.174158

dsite12_2020 | .8569214 .6724481 1.27 0.203 -.4610526 2.174895

dsite13_2020 | .2287153 .5020688 0.46 0.649 -.7553216 1.212752

dsite14_2020 | -.7545422 .8238075 -0.92 0.360 -2.369175 .8600909

_cons | -.7548479 .0768236 -9.83 0.000 -.9054194 -.6042764

-------------+----------------------------------------------------------------

/lnalpha | -1.348746 .2152162 -1.770562 -.9269304

-------------+----------------------------------------------------------------

alpha | .2595654 .0558627 .1702372 .3957667

------------------------------------------------------------------------------

LR test of alpha=0: chibar2(01) = 39.89 Prob >= chibar2 = 0.000

Source | SS df MS Number of obs = 1,708

-------------+---------------------------------- F(27, 1680) = 196.79

Model | 1929320.25 27 71456.3056 Prob > F = 0.0000

Residual | 610026.311 1,680 363.110899 R-squared = 0.7598

-------------+---------------------------------- Adj R-squared = 0.7559

Total | 2539346.56 1,707 1487.60783 Root MSE = 19.055

------------------------------------------------------------------------------

stringency | Coef. Std. Err. t P>|t| [95% Conf. Interval]

-------------+----------------------------------------------------------------

yearC | 65.12287 .9221588 70.62 0.000 63.31417 66.93157

dsite2 | 6.71e-13 2.439803 0.00 1.000 -4.785373 4.785373

dsite3 | 6.58e-13 2.439803 0.00 1.000 -4.785373 4.785373

dsite4 | 6.93e-13 2.439803 0.00 1.000 -4.785373 4.785373

dsite5 | 6.66e-13 2.439803 0.00 1.000 -4.785373 4.785373

dsite6 | 5.448525 2.439803 2.23 0.026 .6631514 10.2339

dsite7 | 17.30828 2.439803 7.09 0.000 12.52291 22.09365

dsite8 | 17.30828 2.439803 7.09 0.000 12.52291 22.09365

dsite9 | 7.361558 2.439803 3.02 0.003 2.576184 12.14693

dsite10 | 4.490984 2.439803 1.84 0.066 -.2943896 9.276357

dsite11 | 11.86934 2.439803 4.86 0.000 7.083971 16.65472

dsite12 | 3.065984 2.439803 1.26 0.209 -1.71939 7.851357

dsite13 | 7.72459 2.439803 3.17 0.002 2.939217 12.50996

dsite14 | 5.766967 2.439803 2.36 0.018 .981594 10.55234

dsite2_2020 | 5.04e-12 4.879606 0.00 1.000 -9.570747 9.570747

dsite3_2020 | 5.09e-12 4.879606 0.00 1.000 -9.570747 9.570747

dsite4_2020 | 5.21e-12 4.879606 0.00 1.000 -9.570747 9.570747

dsite5_2020 | 5.04e-12 4.879606 0.00 1.000 -9.570747 9.570747

dsite6_2020 | 10.89705 4.879606 2.23 0.026 1.326303 20.4678

dsite7_2020 | 34.61656 4.879606 7.09 0.000 25.04581 44.1873

dsite8_2020 | 34.61656 4.879606 7.09 0.000 25.04581 44.1873

dsite9_2020 | 14.72312 4.879606 3.02 0.003 5.152368 24.29386

dsite10_2020 | 8.981967 4.879606 1.84 0.066 -.5887792 18.55271

dsite11_2020 | 23.73869 4.879606 4.86 0.000 14.16794 33.30944

dsite12_2020 | 6.131967 4.879606 1.26 0.209 -3.438779 15.70271

dsite13_2020 | 15.44918 4.879606 3.17 0.002 5.878434 25.01993

dsite14_2020 | 11.53393 4.879606 2.36 0.018 1.963188 21.10468

_cons | 32.56143 .4610794 70.62 0.000 31.65708 33.46579

------------------------------------------------------------------------------

| Estimate Std Err P>|z| [95% Conf Interval]

-------------+-------------------------------------------------------

cde | 1.4080427 .15364719 0.026 1.0419063 1.9028429

nie | .48736124 .11321701 0.000 .39037206 .60844769

te | .68622542 .11808594 0.001 .54443999 .86493522

cde:controlled direct effect, nie:natural indirect effect, te:total effect

xxxxxxx

------------------------------------------------------------------------------

| Observed Bootstrap

| Coef. Bias Std. Err. [95% Conf. Interval]

-------------+----------------------------------------------------------------

cde | 1.4080427 -.065512 .68808132 .3752612 5.361488 (BC)

nie | .48736124 .0043984 .05563412 .3897991 .6030075 (BC)

te | .68622542 -.0334326 .32906193 .1861611 2.548415 (BC)

------------------------------------------------------------------------------

(BC) bias-corrected confidence interval

Note: One or more parameters could not be estimated in 7 bootstrap replicates;

standard-error estimates include only complete replications.

.

end of do-file

.
