# Supplementary material for: Lockdown stringency and paediatric self-harm presentations during COVID-19 pandemic: retrospective cohort study
Source: BJPsych Open. 2022 Mar 24;8(2):e75. doi: 10.1192/bjo.2022.41 (PMC8963968; doi:10.1192/bjo.2022.41)
Supplement: Supplementary file 1 [file bjosup.zip › S2056472422000412sup001.docx]

**Supplementary material 1**

Summary of site categorisation

| **Hospital/NHS trust** | **Site cluster** | **Country** |
| --- | --- | --- |
| Medical University of Vienna | Vienna | Austria |
| Vadaskert Alapitvany | Budapest | Hungary |
| University Hospital of Cagliari | Cagliari | Italy |
| University of Turin - Child & Adolescent Neuropsychiatry | Turin |  |
| Sultan Qaboos University Hospital, Al-Massara Hospital | Muscat | Oman |
| Temple Street Children’s University Hospital | Dublin | Republic of Ireland |
| Children's Health Ireland at Crumlin |  |  |
| Clinic for Neurology and Psychiatry for Children and Youth | Belgrade | Serbia |
| Acıbadem Mehmet Ali Aydınlar University Medical Faculty | Istanbul | Turkey |
| Rashid Hospital, Dubai Hospital, and Latifa hospital | Dubai | United Arab Emirates |
| Royal Edinburgh Hospital | Edinburgh | Scotland |
| Royal Infirmary of Edinburgh |  |  |
| Royal Hospital for Sick Children |  |  |
| St John’s Hospital | Livingston |  |
| Central and North West London NHS Foundation Trust | London | England |
| Kings College Hospital |  |  |
| St Thomas’ Hospital |  |  |
| Croydon University Hospital |  |  |
| Lewisham University Hospital |  |  |
| Berkshire Healthcare NHS Foundation Trust | Home Counties |  |
| Lister Hospital |  |  |
| Watford General Hospital |  |  |
| Royal Manchester Children's Hospital | Manchester |  |
